# Subacute Combined Degeneration of the Spinal Cord Secondary to Nitrous Oxide Abuse

**DOI:** 10.7759/cureus.35341

**Published:** 2023-02-22

**Authors:** Aamir Khan, Ayesha Zafar, Hira Hamid, Bilal Ahmad

**Affiliations:** 1 Neurology, Queen Elizabeth Hospital Birmingham, Birmingham, GBR; 2 Neurology, Medical Teaching Institute Lady Reading Hospital, Peshawar, PAK; 3 Neurology, Lady Reading Hospital Peshawar, Peshawar, PAK; 4 Internal Medicine, Lady Reading Hospital Peshawar, Peshawar, PAK

**Keywords:** vitamin b12, mri, methionine, nitrous oxide, scid

## Abstract

Here, we report a case of subacute combined degeneration (SCD) of the spinal cord in the setting of nitrous oxide poisoning seen at the Medical Ward, Queen Elizabeth Hospital Birmingham Our patient was a 28-year-old lady who presented with impaired sensations in the lower limbs and difficulty walking for approximately one and a half months. Her clinical symptoms did not match common neurological conditions. Upon detailed history, she revealed that she had been frequently using nitrous oxide recreationally for several years. Although her baseline investigations were normal, her magnetic resonance imaging (MRI) of the spine showed bilateral symmetrical T2 hyperintense signal changes in the dorsal columns extending from C2 to C6 spinal segment. Based on history, clinical findings, and MRI of the cervical spine, the diagnosis of SCD of the spinal cord was made, and her symptoms fully resolved with treatment.

## Introduction

Nitrous oxide is commonly used as an anesthetic in dental procedures. However, its increasing recreational use can cause vitamin B12 deficiency which can cause peripheral neuropathies and myelopathies leading to severe neurological consequences.

Here, we report a case of subacute combined degeneration (SCD) of the spinal cord in the setting of nitrous oxide poisoning which presented to the Medical Ward, Queen Elizabeth Hospital Birmingham.

## Case presentation

**Figure 1:**
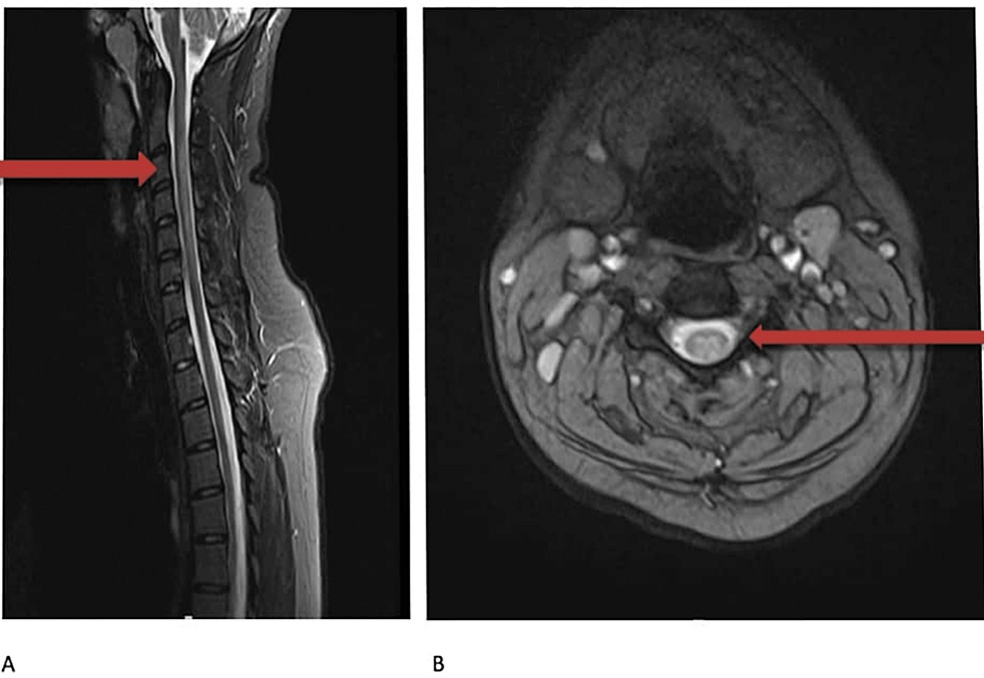
(A, B) MRI of the cervical spine demonstrating bilateral symmetrical T2 high signal changes in the dorsal columns extending from C2 to C6 level.

A 28-year-old lady was admitted to our medical unit with complaints of tingling and numbness in both of her hands and feet for a month. She admitted to a history of recreational use of nitrous oxide inhalation, smoking cannabis/marijuana, and consuming amphetamine. She had a power of 5/5 in all limbs with diminished reflexes and impaired pain sensation in glove and stocking distribution. There was an absent joint position and vibration sense. The routine blood examination including full blood counts and liver and kidney functions were normal. Vitamin B12 level was low (188 pg/mL) with normal homocysteine and methylmalonic acid (MMA) levels. The patient was seen by the neurology team and was advised to undergo magnetic resonance imaging (MRI) of the brain and whole spine with contrast. The brain MRI was normal. However, MRI of the spine showed bilateral symmetrical T2 hyperintense signal changes in the dorsal column extending from C2 to C6 level which is typical of SCD of the cord, as described in sagittal (Figure 1A) and axial views of the cord (Figure 1B). No significant cord swelling or contrast enhancement was reported. The thoracic cord appeared unremarkable.

She was managed as a case of SCD of the spinal cord likely due to functional vitamin B12 deficiency secondary to nitrous oxide inhalation. She was started on intramuscular vitamin B12 treatment as per British National Formulary (BNF) guidelines and her symptoms started to resolve. At the one-month office follow-up, the patient reported overall symptomatic improvement. She was discharged on treatment with the advice to stop nitrous oxide use and complete vitamin B12 injections as per the BNF protocol. Her neurophysiological studies showed reduced peroneal motor responses with prolonged distal latencies which supported a predominantly demyelinating neuropathy. Also seen was a slowing of some motor and sensory conduction velocities, mainly in the lower limbs, and borderline f-wave responses from tibial nerves. There was mildly reduced recruitment and no signs of active denervation, which is consistent with the characteristics of demyelinating neuropathy. The diagnosis of SCD of the spinal cord was established based on the neurophysiological and radiological findings.

## Discussion

SCD is characterized by demyelination of the spinal cord arising due to a deficiency of vitamin B12, which can cause symptoms such as spastic paraparesis and impaired vibration and proprioception sense, leading to sensory ataxia and difficulty in walking. The symptoms occur due to the development of lesions in the cord’s lateral corticospinal tracts and dorsal columns, respectively [1]. In addition to this, the patient can have psychosis and memory impairment [2]. The laboratory findings include macrocytic anemia and hypersegmented neutrophils. The most common cause of vitamin B12 deficiency is pernicious anemia, with other causes including the absence of the terminal ileum and a strict vegetarian diet. Excessive inhalation of nitrous oxide is a rare cause of vitamin B12 deficiency. Prolonged/frequent use of nitrous oxide can convert the active bivalent form of vitamin B12 to an inactive monovalent form, leading ultimately to an indirect depletion of vitamin B12 in the body [3].

Nitrous oxide has various conventional medical applications, such as its use as an anesthetic agent during dental and surgical procedures. However, due to its easy availability over the counter, it can sometimes be abused. Various studies have reported an association between chronic recreational uses of nitrous oxide and subsequent gait disorder by the deactivation of vitamin B12 and its downstream effect on methionine synthesis [4]. It has been shown that in patients with SCD vitamin B12 level was very low; however, nitrous oxide can cause neurological deficits in patients with normal vitamin B12 level as well. In different clinical settings, the recreational use of vitamin B12 is linked with SCD [5].

In cases of nitrous oxide neurotoxicity, similar to classic SCD, patients typically report symptoms such as limb numbness, limb weakness, and gait disturbance. However, unlike other causes of vitamin B12 deficiency, nitrous oxide-induced vitamin B12 deficiency often presents with a normal hematological pattern [6]. Age and disease course are the main prognostic factors in patients with SCD, but in both anesthetic and recreational use, worsened clinical manifestations are not expected to be associated with classic anemia, low levels of serum vitamin B12, or MRI abnormalities in the spinal cord.

In treating patients with SCD, usually, abstinence from nitrous oxide with parenteral injections of hydroxocobalamin (vitamin B12 ) lead to greater symptomatic improvement [7]. Previous studies have reported significant improvement in patients with parental hydroxocobalamin in one month and on a three-month follow-up [7].

The course of symptom resolution is not associated with sex, hemoglobin level, serum vitamin B12, or MRI manifestations at the time of admission and at follow-up visits [8]. However, young patients with early presentation and a short duration of the disease have better prognoses. In our case, similarly, the patient presented early at a young age and was managed with abstinence from nitrous oxide and parenteral hydroxocobalamin injection with dramatic improvement in the symptoms. However, in our case, the MMA level was normal compared to the raised level reported in other studies [9].

## Conclusions

Nitrous oxide recreational abuse can lead to permanent neurological manifestations and early diagnosis and treatment can be dramatic in long-term prognosis. Clinical staff must be aware of the various types of presentations of neurotoxicity related to nitrous oxide abuse, and chronic nitrous oxide abuse should be the part of differential of patients who present with atypical neurological symptoms.
